# Pathways between Socioeconomic Disadvantage and Childhood Growth in the Scottish Longitudinal Study, 1991–2001

**DOI:** 10.1371/journal.pone.0164853

**Published:** 2016-10-13

**Authors:** Richard J. Silverwood, Lee Williamson, Emily M. Grundy, Bianca L. De Stavola

**Affiliations:** 1 Department of Medical Statistics, London School of Hygiene and Tropical Medicine, London, United Kingdom; 2 Centre for Statistical Methodology, London School of Hygiene and Tropical Medicine, London, United Kingdom; 3 School of GeoSciences, University of Edinburgh, Edinburgh, United Kingdom; 4 Ageing, Lifecourse and Population Health Analysis Research Unit, Department of Social Policy, London School of Economics and Political Science, London, United Kingdom; Hunter College, UNITED STATES

## Abstract

Socioeconomically disadvantaged children are more likely to be of shorter stature and overweight, leading to greater risk of obesity in adulthood. Disentangling the mediatory pathways between socioeconomic disadvantage and childhood size may help in the development of appropriate policies aimed at reducing these health inequalities. We aimed to elucidate the putative mediatory role of birth weight using a representative sample of the Scottish population born 1991–2001 (n = 16,628). Estimated height and overweight/obesity at age 4.5 years were related to three measures of socioeconomic disadvantage (mother’s education, Scottish Index of Multiple Deprivation, synthetic weekly income). Mediation was examined using two approaches: a ‘traditional’ mediation analysis and a counterfactual-based mediation analysis. Both analyses identified a negative effect of each measure of socioeconomic disadvantage on height, mediated to some extent by birth weight, and a positive ‘direct effect’ of mother’s education and Scottish Index of Multiple Deprivation on overweight/obesity, which was partly counterbalanced by a negative ‘indirect effect’. The extent of mediation estimated when adopting the traditional approach was greater than when adopting the counterfactual-based approach because of inappropriate handling of intermediate confounding in the former. Our findings suggest that higher birth weight in more disadvantaged groups is associated with reduced social inequalities in height but also with increased inequalities in overweight/obesity.

## Introduction

The existence of social inequalities in health is well established, with poor health disproportionately burdening those of lower socioeconomic status [1]. There is evidence to suggest that the early years of development play a critical role in the creation of socioeconomic health inequalities which are maintained into adulthood [2, 3]. Socioeconomic differences in childhood growth are therefore an important area of research. Such socioeconomic differences in childhood growth may ultimately be tackled by a reduction in socioeconomic inequality itself, but a better understanding of the mediatory pathways through which the effects of deprivation act might give rise to alternative, possibly more achievable, interventions.

In this paper we consider childhood growth in terms of age-specific height and overweight/obesity (hereafter ‘overweight’). Height in childhood is often considered as a marker of development, with long-term consequences for future health [4]. In Britain it has been consistently found to be lower in children of greater socioeconomic disadvantage [5–10]. As well as affecting the quality of life of young people [11], childhood overweight is associated with adulthood overweight [12, 13], is an established risk factor for diabetes [14] and cardiovascular disease [15], and has been found to be associated with several adult cancers [16]. The prevalence of childhood overweight continues to increase in the UK, particularly among children of greater socioeconomic disadvantage [7, 8, 17–29].

The association between lower birth weight and socioeconomic disadvantage has been extensively documented [10, 30, 31]. Greater weight at birth has been shown to be predictive of increased height [5, 32] and body mass index (BMI) [24, 33, 34] in childhood. Although there is some debate about the causal nature of these relationships, particularly since the associations were drawn from observational studies, several studies have found strong associations between birth weight and height or obesity that were robust to adjustment for several putative confounders [24, 32, 33, 34]. Birth weight is thus a plausible mediator in the associations observed between socioeconomic disadvantage and childhood height and overweight. However, few studies have explicitly examined this [35].

The aim of the current study was to elucidate the putative mediatory role of birth weight in the relationship between socioeconomic disadvantage and childhood growth in terms of height and overweight using a large-scale representative sample of the Scottish population. Furthermore, we aimed to provide a comparison of two different methods for examining mediation.

## Materials and Methods

### Participants

The Scottish Longitudinal Study (SLS) is an anonymised record linkage study covering a 5.3% sample of the Scottish population, selected using 20 birth dates [36]. In contains linked Census and vital registration data from 1991 onwards and, with appropriate permissions, can be linked to health data.

The sample considered for the present analysis are the 43,286 SLS members born from 1991 to 2001. Many analysis variables were from data sources other than the Census: birth registrations, Scottish Morbidity Records, maternity records and Child Health Systems Programme (CHSP) Pre-School data.

The SLS contains no identifiable individual level data, and data are derived from linkages that are anonymised prior to analysis by the research team. Ethical approval for the study was granted by the London School of Hygiene and Tropical Medicine Research Ethics Committee (reference 6418) and by the National Health Service National Services Scotland Privacy Advisory Committee for approval of the health data linkage.

### Measures

#### Socioeconomic disadvantage

Three different measures of socioeconomic disadvantage were considered as the exposures of interest: mother’s education; an ecological measure using the Scottish Index of Multiple Deprivation (SIMD); and a synthetic measure of weekly household income. These variables were chosen to cover different domains of socioeconomic disadvantage (individual-level education, area-level multiple deprivation, and household-level occupation/income). Categorical measures of each socioeconomic disadvantage variable were used in the analyses to allow for potential non-linear effects.

Mother’s education was derived from 2001 Census data and categorised as: ‘no qualifications’, ‘GCSE or equivalent’ (exams usually taken at age 15/16 years), ‘A-level or equivalent’ (exams usually taken at age 17/18 years), ‘degree or equivalent’.

The SIMD defines small area concentrations of multiple deprivation within Scotland across several different domains [37]. SIMD values were derived using the postcode recorded on the birth record and reflected the SIMD level for that area in 2001. SIMD was analysed in quarters of the observed distribution.

Income data are often missing or poorly measured in observational studies due to their inherent complexity and sensitivity. One solution, recently proposed by Clemens and Dibben [38], is to derive a synthetic measure of weekly income using observed data on occupation. We used the occupation of the SLS member’s mother and father reported on the birth record of the SLS member to derive synthetic weekly income using this approach [38]. An estimate of income was made for parents not in paid employment on the basis of typical social security payments. Household income was calculated as the sum of the mother’s and father’s estimated incomes, and an income equalisation multiplier of 1.6 was applied for single mothers. The final synthetic weekly household income was analysed in quarters of the observed distribution.

#### Anthropometric measures

The CHSP Pre-School is a programme of pre-school child health reviews carried out by nurses and health visitors in Scotland at a series of designated ages. The CHSP Pre-School was established in 1991 but had a phased implementation across the 15 Health Boards in Scotland. Ten Health Boards (Ayrshire & Arran, Borders, Argyll & Clyde, Fife, Greater Glasgow, Lanarkshire, Lothian, Tayside, Forth Valley and Dumfries & Galloway) had implemented the CHSP Pre-School by 2000 [39] and were included in the present analysis.

Length/height, weight and age data were extracted from the CHSP Pre-School records at the following approximate ages: 6–8 weeks, 8–9 weeks, 21–24 months, 39–42 months and 48 months. It should be noted that in those Health Boards where the implementation of the CHSP Pre-School was relatively late (e.g. Dumfries and Galloway in 2000), some or all of the anthropometric measurements were not available for SLS members born early in the period being considered.

#### Potential mediator

The mediator of interest was birth weight (<2.50 kg, 2.50–2.99 kg, 3.00–3.49 kg, 3.50+ kg; derived from maternity records). Birth weight was considered as a categorical variable in the analyses to allow for potential non-linear effects.

#### Potential confounders

Several background confounding factors were considered: sex, year of birth (1991–1994, 1995–1999, 2000–2005), Health Board (10 regions) and ethnicity (white, non-white).

#### Potential intermediate confounders

Intermediate confounders are common causes of the mediator and outcome that are themselves causally affected by the exposure. We considered maternal age at the birth of the study child (<20, 20–24, 25–29, 30–34, 35+ years) and parity prior to the birth of the study child (0, 1, 2+; derived from maternity records) as intermediate confounders

**Fig 1:**
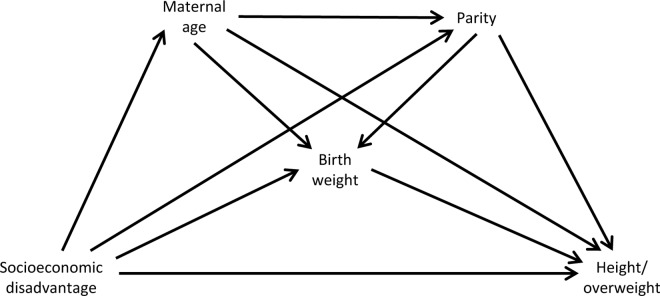
Assumed causal diagram for the two outcomes of interest (height and overweight/obesity at age 4.5 years). Background confounders (sex, year of birth, Health Board and ethnicity) are omitted to aid clarity.

The hypothesised causal relationships between the analysis variables are shown in Fig 1.

### Statistical methods

A two-stage modelling approach was used to first define the outcomes and then perform mediation analysis. In the first stage the repeated anthropometric measurements were modelled to predict height and weight at the age of 4.5 years. This age was chosen because it is the approximate mean age at the final CHSP Pre-School measurement. In the second stage, mediation of the effect of the different measures of socioeconomic disadvantage on height and overweight at age 4.5 years by birth weight was assessed using two different approaches: i) ‘traditional’ mediation analysis and ii) counterfactual-based mediation analysis.

#### Stage 1: Growth modelling

Full details of the growth modelling are given in S1 Appendix. Briefly, the repeated measurements of height and weight were modelled separately in males and females using mixed effects Berkey-Reed models [40, 41]. All subjects with at least one valid growth measurement were included in the modelling, assuming missingness was at random [42]. The fitted models were used to predict subject-specific height (cm) and weight (kg) at age 4.5 years, with predicted BMI at age 4.5 years derived from the predicted height and weight values (weight (kg)/height (m)^2^). The age- and sex-specific international overweight cut-offs of Cole et al [43] (17.47 kg/m^2^ for males and 17.19 kg/m^2^ for females) were then used to define overweight at age 4.5 years (a binary variable) from the predicted BMI. Predicted height and predicted overweight status were the outcomes of interest to be used in the second stage.

#### Stage 2: Mediation analysis

This step was achieved using two different approaches: i) ‘traditional’ mediation analysis and ii) counterfactual-based mediation analysis

A frequently used approach to mediation analysis, referred to here as a ‘traditional’ mediation analysis and elsewhere as the ‘difference method’ [44], is that popularised in a 1986 paper by Baron and Kenny [45]. In this approach two separate regression models are fitted: the first relating the outcome to the exposure (and any confounders) and the second relating the outcome to the exposure and the mediator (and any confounders). In the first model the estimated parameter for the exposure is interpreted as the ‘total effect’ of the exposure on the outcome, in the second model it is interpreted as the effect of the exposure on the outcome not mediated by the mediator (the ‘direct effect’). The difference between the ‘total effect’ and the ‘direct effect’ can then be calculated to give the effect of the exposure on the outcome mediated by the mediator (the ‘indirect effect’). Estimation is carried out under the standard assumptions of linear regression, i.e. that the error terms in the regression models are uncorrelated with the explanatory variables and have conditional means of zero.

However, this approach is only valid for linear models which do not include exposure-mediator interactions or intermediate confounding [46–48]. We adopt it here (despite the presence of intermediate confounding and using a generalised linear model for the binary outcome) as a comparator to the more general counterfactual–based approach (see below). Because the presence of intermediate confounding means we do not expect to obtain unbiased estimates of the direct and indirect effects using the traditional approach, we refer to the ‘direct effect’ and ‘indirect effect’ (in inverted commas) when discussing results of this analysis.

For the traditional approach, each derived growth outcome was related to the different indicators of socioeconomic disadvantage using linear or logistic regression (for height and overweight respectively). For each socioeconomic disadvantage measure the following two models were fitted using maximum likelihood estimation:

- Adjusted for background confounders (sex, year of birth, Health Board and ethnicity);
- Additionally adjusted for the mediator, birth weight.

The regression coefficient for socioeconomic disadvantage in the first fitted model thus gives the estimated ‘total effect’, and the same parameter in the second fitted model gives the estimated ‘direct effect’, with the difference between them giving the estimated ‘indirect effect’. Model specifications were investigated by separately testing the significance of the interaction between the exposure of interest and each covariate (confounders and birth weight) using Wald tests. The ‘proportion mediated’ was then calculated (on the log-odds ratio (OR) scale for overweight models).

According to the literature, we would expect both a negative direct effect of socioeconomic disadvantage on height and a negative indirect effect of socioeconomic disadvantage on height, resulting in ‘consistent mediation’ [49]. On the other hand for overweight we would expect a positive direct effect and negative indirect effect, i.e. ‘inconsistent mediation’ [49]. The proportion mediated was thus calculated as the estimated ‘indirect effect’ divided by the estimated total effect for consistent mediation, and as the absolute estimated ‘indirect effect’ divided by the sum of the absolute estimated ‘indirect effect’ and the absolute estimated ‘direct effect’ for inconsistent mediation [50].

To account for between-subject differences in the precision of the estimated growth outcomes the analyses were weighted by the inverse of the approximate variance of the predicted growth outcome. A robust standard error estimator was used.

As noted above, traditional mediation analysis has several limitations. In addition to its restriction to linear models, of particular concern in the present study is that intermediate confounding cannot be appropriately handled. As shown in Fig 1, maternal age and parity are presumed intermediate confounders in the present setting.

An alternative approach that can appropriately handle non-linear models and intermediate confounding has been proposed in the causal inference literature. It uses counterfactual definitions of direct and indirect effects allowing for generality and greater formality than the traditional approach [51]. Among several possible definitions proposed in this framework we consider here the ‘natural direct effect’ (NDE) and ‘natural indirect effect’ (NIE) [46, 52]. Concerns regarding the relevance of natural effect estimates have been expressed, particularly in regards to the ‘cross-world’ assumption often invoked to identify these estimands [53]. We acknowledge that this assumption could not be verified even in a hypothetical experiment. However, our focus is on partitioning the effect of an exposure into separate pathways; controlled effects, which are often suggested as alternative mediation estimands, cannot achieve this. The interpretation of NDE/NIE (what would happen if we could disable the pathway from the exposure to the mediator, and hence to study the strength of the mechanism that involves birth weight) is more appropriate for this type of exploratory mediation analysis.

Let *X*_*i*_ be socioeconomic disadvantage, *Y*_*i*_ be height at age 4.5 years, *M*_*i*_ be birth weight, *C*_*i*_ be the set of background confounders (sex, year of birth, Health Board and ethnicity) and *L*_*i*_ be the set of intermediate confounders (maternal age and parity) for child *i*. Let *Y*_*i*_(*x*) be the value that *Y*_*i*_ would take if *X*_*i*_ had been set (possibly counter to fact) to the value *x*, *Y*_*i*_(*x*,*m*) be the value that *Y*_*i*_ would take if *X*_*i*_ and *M*_*i*_ had been set to the values *x* and *m*, and *M*_*i*_(*x*) be the value that *M*_*i*_ would take if *X*_*i*_ had been set to the value *x*.

The ‘total causal effect’ (TCE) of *X* on *Y*, conditional on *C* = *c*, expressed as a mean difference comparing *X* = x* to *X* = *x* is

$$TCE\;(c,x,x^{*})=E(Y_{i}(x^{*})\;|\;C_{i}=c)-E(Y_{i}(x)\;|\;C_{i}=c),$$

the NDE of *X* on *Y*, conditional on *C* = *c*, expressed as a mean difference comparing *X* = x* to *X* = *x* is

$$NDE(c,x,x^{*})=E(Y_{i}(x^{*},M_{i}(x))\;|\;C_{i}=c)-E(Y_{i}(x,M_{i}(x))\;|\;C_{i}=c),$$

and the NIE of *X* on *Y* conditional on *C* = *c*, expressed as a mean difference comparing *X* = x* to *X* = *x* is

$$NIE\;(c,x,x^{*})=E(Y_{i}(x^{*},M_{i}(x^{*}))\;|\;C_{i}=c)-E(Y_{i}(x^{*},M_{i}(x))\;|\;C_{i}=c).$$

The TCE, NDE and NIE can be analogously defined on the OR scale, as shown in S2 Appendix.

Identification of these effects requires certain assumptions. Those usually invoked are non-interference, consistency, and no unmeasured confounding of the exposure-mediator, exposure-outcome and mediator-outcome relationships [52, 54]. Additionally, because of the presence of intermediate confounders, a version of the cross-world assumption is required. In our applications this took the form of no non-linearities in the effect of the intermediate confounders on the outcome [48].

Estimation was performed via parametric G-computation using Monte Carlo simulation under the additional assumption of correct model specification [54–56]. The G-computation procedure is described in S3 Appendix. Using this approach the TCE of each socioeconomic disadvantage variable on height and overweight at age 4.5 years was decomposed into a NDE (not via birth weight) and a NIE (via birth weight) on the mean difference (height) or OR (overweight) scale.

Confidence intervals (CIs) were obtained using a bootstrap approach with 1000 draws. The proportion mediated was calculated using the same approach as in the traditional mediation analysis.

Analyses were restricted to complete records after assessing the extent of the likely selection bias. All analyses were conducted using Stata version 12 [57]. Estimation by G-computation was performed using the ‘gformula’ command [55].

## Results

Growth data availability by age and by subject is shown in S1 Table and S2 Table respectively. 24,703 subjects with at least one valid height observation had height models fitted and height at age 4.5 years predicted. 24,509 subjects with fitted height and weight at age 4.5 years had BMI (and then overweight) at age 4.5 years derived. Of the subjects with derived height and/or overweight at age 4.5 years, 16,628 had complete data on birth weight, all the potential confounders and one or more of the socioeconomic disadvantage measures. The distributions of all the analysis variables are given in Table 1. The distributions of variables in the analysis sample were in general very similar to those in the sample overall (S3 Table). One exception was the relative preponderance of later births in the analysis (due to the phased implementation of the CHSP Pre-School). The distributions of height and overweight by each explanatory variable are given in S4 Table.

**Table 1:** Distributions of explanatory variables for study members included in the analysis^a^, Scottish Longitudinal Study, United Kingdom, 1991–2001 (n = 16,628).

Variable	N	n (%)
Mother’s education	15,031	
No qualifications		2,330 (15.5)
GCSE or equivalent		5,355 (35.6)
A-level of equivalent		3,879 (25.8)
Degree or equivalent		3,467 (23.1)
Scottish Index of Multiple Deprivation quarter	16,588	
1 (Most deprived)		3,970 (23.9)
2		4,291 (25.9)
3		3,967 (23.9)
4 (Least deprived)		4,360 (26.3)
Synthetic income quarter	16,627	
1 (lowest)		4,552 (27.4)
2		4,618 (27.8)
3		4,429 (26.6)
4 (highest)		3,028 (18.2)
Sex	16,628	
Male		8,578 (51.6)
Female		8,050 (48.4)
Year of birth	16,628	
1991–1994		5,323 (32.0)
1995–1998		5,642 (33.9)
1999–2001		5,663 (34.1)
Health Board	16,628	
Ayrshire & Arran		1,126 (6.8)
Borders		483 (2.9)
Argyll & Clyde		1,959 (11.8)
Fife		1,441 (8.7)
Greater Glasgow		3,141 (18.9)
Lanarkshire		2,499 (15.0)
Lothian		3,295 (19.8)
Tayside		1,568 (9.4)
Forth Valley		905 (5.4)
Dumfries & Galloway		211 (1.3)
Ethnicity	16,628	
White		16,174 (97.3)
Non-white		454 (2.7)
Birth weight (kg)	16,628	
<2.50		807 (4.9)
2.50–2.99		2,450 (14.7)
3.00–3.49		5,959 (35.8)
3.50+		7,412 (44.6)
Mother’s age at the birth of the study child (years)	16,628	
<20		1,123 (6.8)
20–24		2,869 (17.3)
25–29		5,427 (32.6)
30–34		5,031 (30.3)
35+		2,178 (13.1)
Mother’s parity prior to the birth of the study child	16,628	
0		7,678 (46.2)
1		5,879 (35.4)
2+		3,071 (18.5)

^a^Complete data on birth weight, all the potential confounders and one or more of the socioeconomic disadvantage measures.

Source: Scottish Longitudinal Study linked to maternity records and Child Health Systems Programme Pre-School data

### Traditional mediation analysis

There was evidence of a strong, graded association between all measures of socioeconomic disadvantage and predicted height at age 4.5 years in the confounder adjusted models with, for example, the most disadvantaged category for mother’s education estimated to be associated with a 0.98 cm (95% CI 0.75, 1.22) lower height relative to the least disadvantaged category (Table 2). The estimated associations were attenuated on additional adjustment for birth weight. Comparing the most disadvantaged category to the least disadvantaged category the estimated proportion mediated was 43% for maternal education, 40% for SIMD and 88% for synthetic income (Table 2).

**Table 2:** Estimated regression coefficients for associations between measures of socioeconomic disadvantage and predicted height at age 4.5 years, Scottish Longitudinal Study, United Kingdom, 1991–2001.

	Confounder adjusted			Additionally adjusted for birth weight			
	Coeff	95% CI	*P* overall (*P* trend)	Coeff	95% CI	*P* overall (*P* trend)	Proportion mediated
Mother’s education (n = 15,031)							
No qualifications	-0.98	-1.22, -0.75	<0.001	-0.56	-0.79, -0.33	<0.001	0.43
GCSE or equivalent	-0.50	-0.69, -0.32		-0.28	-0.46, -0.10		0.44
A-level of equivalent	0.02	-0.17, 0.22		0.11	-0.08, 0.30		-
Degree or equivalent	0.00	(ref)		0.00	(ref)		
SIMD quarter (n = 16,588)							
1 (most deprived)	-0.91	-1.10, -0.72	<0.001	-0.55	-0.73, -0.36	<0.001	0.40
2	-0.44	-0.62, -0.25	(<0.001)	-0.27	-0.45, -0.09	(<0.001)	0.39
3	-0.25	-0.44, -0.06		-0.19	-0.37, 0.00		0.24
4 (least deprived)	0.00	(ref)		0.00	(ref)		
Synthetic income quarter (n = 16,627)							
1 (lowest)	-0.75	-0.97, -0.54	<0.001	-0.09	-0.32, 0.13	0.001	0.88
2	-0.59	-0.80, -0.38		-0.14	-0.35, 0.07		0.76
3	-0.04	-0.24, 0.17		0.20	0.01, 0.40		-
4 (highest)	0.00	(ref)		0.00	(ref)		

CI, confidence interval; SIMD, Scottish Index of Multiple Deprivation.

All models adjusted for sex, year of birth, Health Board and ethnicity.

Source: Scottish Longitudinal Study.

Table 3 shows the estimated ORs for the associations between each measure of socioeconomic disadvantage and predicted overweight at age 4.5 years. There was only weak evidence of associations for maternal education and SIMD in the confounder adjusted models with, for example, the most disadvantaged category for mother’s education estimated to be associated with 26% (95% CI 5, 52) higher odds of overweight relative to the least disadvantaged category. These associations were markedly amplified on additional adjustment for birth weight. Comparing the most disadvantaged category to the least disadvantaged category the estimated proportion mediated was 27% for maternal education, 25% for SIMD and 35% for synthetic income.

**Table 3:** Estimated odds ratios for associations between measures of socioeconomic disadvantage and predicted overweight status at age 4.5 years, Scottish Longitudinal Study, United Kingdom, 1991–2001.

	Confounder adjusted			Additionally adjusted for birth weight			
	OR	95% CI	*P* overall (*P* trend)	OR	95% CI	*P* overall (*P* trend)	Proportion mediated
Mother’s education (n = 14,935)							
No qualifications	1.26	1.05, 1.52	0.08	1.45	1.20, 1.75	0.001	0.27
GCSE or equivalent	1.15	0.99, 1.34	(0.02)	1.23	1.06, 1.44	(<0.001)	0.25
A-level of equivalent	1.17	1.00, 1.37		1.20	1.03, 1.41		0.12
Degree or equivalent	1.00	(ref)		1.00	(ref)		
SIMD quarter (n = 16,477)							
1 (most deprived)	1.22	1.04, 1.42	0.05	1.35	1.16, 1.58	0.001	0.25
2	1.19	1.02, 1.38	(0.01)	1.24	1.07, 1.44	(<0.001)	0.16
3	1.18	1.01, 1.37		1.20	1.03, 1.40		0.08
4 (least deprived)	1.00	(ref)		1.00	(ref)		
Synthetic income quarter (n = 16,516)							
1 (lowest)	1.09	0.92, 1.29	0.53	1.20	1.00, 1.45	0.12	0.35
2	1.02	0.87, 1.21	(0.25)	1.09	0.92, 1.29	(0.03)	0.44
3	0.97	0.83, 1.14		1.01	0.86, 1.18		-
4 (highest)	1.00	(ref)		1.00	(ref)		

CI, confidence interval; OR, odds ratio; SIMD, Scottish Index of Multiple Deprivation.

All models adjusted for sex, year of birth, Health Board and ethnicity.

Source: Scottish Longitudinal Study.

There was no evidence of interactions between the exposures of interest and either birth weight or the confounders in any models.

### Counterfactual-based mediation analysis

The TCEs for each measure of socioeconomic disadvantage estimated by G-computation (Table 4) were very similar to the corresponding estimated regression coefficients in the traditional mediation analysis (confounder adjusted model, Table 2) as would be expected. The estimated NDEs however were more pronounced than their traditional counterparts leading to smaller percentage mediated. Comparing the most disadvantaged category to the least disadvantaged category, the estimated proportion mediated was 20% for maternal education, 20% for SIMD and 16% for synthetic income.

**Table 4:** Estimated mean differences for associations between measures of socioeconomic disadvantage and predicted height at age 4.5 years, Scottish Longitudinal Study, United Kingdom, 1991–2001.

	TCE			NDE (not via birth weight)			NIE (via birth weight)			Proportion mediated
	Mean diff	95% CI	*P*	Mean diff	95% CI	*P*	Mean diff	95% CI	*P*	
Mother’s education (n = 15,031)										
No qualifications	-0.97	-1.19, -0.75	<0.001	-0.77	-0.99, -0.56	<0.001	-0.20	-0.26, -0.14	<0.001	0.20
GCSE or equivalent	-0.47	-0.64, -0.29	<0.001	-0.32	-0.50, -0.15	<0.001	-0.14	-0.19, -0.09	<0.001	0.30
A-level of equivalent	0.01	-0.17, 0.19	0.92	0.09	-0.09, 0.27	0.32	-0.08	-0.13, -0.03	0.002	-
Degree or equivalent	0.00	(ref)		0.00	(ref)		0.00	(ref)		
SIMD quarter (n = 16,588)										
1 (most deprived)	-0.92	-1.09, -0.75	<0.001	-0.74	-0.91, -0.56	<0.001	-0.18	-0.24, -0.13	<0.001	0.20
2	-0.43	-0.61, -0.26	<0.001	-0.33	-0.50, -0.16	<0.001	-0.10	-0.15, -0.05	<0.001	0.24
3	-0.26	-0.43, -0.09	0.003	-0.18	-0.35, -0.02	0.03	-0.07	-0.12, -0.02	0.003	0.28
4 (least deprived)	0.00	(ref)		0.00	(ref)		0.00	(ref)		
Synthetic income quarter (n = 16,627)										
1 (lowest)	-0.69	-0.90, -0.48	<0.001	-0.58	-0.78, -0.37	<0.001	-0.11	-0.16, -0.06	<0.001	0.16
2	-0.54	-0.74, -0.35	<0.001	-0.44	-0.63, -0.25	<0.001	-0.10	-0.15, -0.06	<0.001	0.19
3	0.01	-0.18, 0.20	0.91	0.11	-0.07, 0.30	0.23	-0.10	-0.15, -0.05	<0.001	-
4 (highest)	0.00	(ref)		0.00	(ref)		0.00	(ref)		

CI, confidence interval; NDE, natural direct effect; NIE, natural indirect effect; SIMD, Scottish Index of Multiple Deprivation; TCE, total causal effect.

All models adjusted for sex, year of birth, Health Board and ethnicity.

Source: Scottish Longitudinal Study.

Table 5 reports the estimated NDEs and NIEs for overweight at age 4.5 years (expressed on the OR scale). In each model, in contrast to the results for height, the estimated NDE was less pronounced than its traditional counterpart. Moreover, the estimated NDEs and NIEs were in the opposite direction, as expected under inconsistent mediation, with evidence of mediation by birth weight (absolute proportion mediated in most disadvantaged category: 18–29%).

**Table 5:** Estimated odds ratios for associations between measures of socioeconomic disadvantage and predicted overweight at age 4.5 years, Scottish Longitudinal Study, United Kingdom, 1991–2001.

	TCE			NDE (not via birth weight)			NIE (via birth weight)			Proportion mediated
	OR	95% CI	*P*	OR	95% CI	*P*	OR	95% CI	*P*	
Mother’s education (n = 14,935)										
No qualifications	1.21	1.03, 1.42	0.02	1.28	1.08, 1.50	0.003	0.95	0.94, 0.97	<0.001	0.19
GCSE or equivalent	1.10	0.97, 1.25	0.15	1.15	1.01, 1.31	0.04	0.97	0.96, 0.99	<0.001	0.23
A-level of equivalent	1.16	1.02, 1.33	0.03	1.19	1.04, 1.36	0.01	0.98	0.97, 1.00	0.003	0.11
Degree or equivalent	1.00	(ref)		1.00	(ref)		1.00	(ref)		
SIMD quarter (n = 16,477)										
1 (most deprived)	1.19	1.03, 1.36	0.01	1.25	1.09, 1.43	0.002	0.95	0.94, 0.97	<0.001	0.18
2	1.13	0.99, 1.29	0.08	1.16	1.01, 1.33	0.03	0.97	0.96, 0.99	<0.001	0.15
3	1.12	0.98, 1.28	0.10	1.14	1.00, 1.31	0.06	0.98	0.97, 1.00	0.01	0.12
4 (least deprived)	1.00	(ref)		1.00	(ref)		1.00	(ref)		
Synthetic income quarter (n = 16,516)										
1 (lowest)	1.05	0.90, 1.22	0.56	1.08	0.93, 1.26	0.32	0.97	0.96, 0.98	<0.001	0.29
2	1.02	0.88, 1.17	0.83	1.04	0.90, 1.20	0.56	0.97	0.96, 0.99	<0.001	0.38
3	0.98	0.85, 1.13	0.81	1.01	0.88, 1.16	0.90	0.97	0.96, 0.99	0.001	-
4 (highest)	1.00	(ref)		1.00	(ref)		1.00	(ref)		

CI, confidence interval; NDE, natural direct effect; NIE, natural indirect effect; OR, odds ratio; SIMD, Scottish Index of Multiple Deprivation; TCE, total causal effect.

All models adjusted for sex, year of birth, Health Board and ethnicity.

Source: Scottish Longitudinal Study.

## Discussion

### Main substantive findings

In this large-scale representative sample of the Scottish population we have elucidated the mediatory role of birth weight in the relationship between socioeconomic disadvantage and childhood growth in terms of height and overweight. Using two different approaches (traditional and counterfactual-based mediation analyses) we found broad agreement that: i) there was a strong, graded, negative ‘effect’ of each measure of socioeconomic disadvantage on height at age 4.5 years which was mediated to some extent by birth weight and ii) there was a strong, graded, positive direct ‘effect’ of each measure of socioeconomic disadvantage (with the exception of synthetic income) on overweight at age 4.5 years which was partly masked by inconsistent mediation by birth weight.

These findings suggest that increased birth weight in more disadvantaged groups may be associated with reduced social inequalities in height but also with increased inequalities in overweight. Birth weight could potentially be a modifiable target of intervention through, for example, programmes designed to improve maternal nutrition or deter maternal smoking during pregnancy [58, 59, 60].

### Main methodological findings

Several assumptions must hold in order for us to obtain unbiased estimates of the NDE and NIE using G-computation, in particular: i) no unmeasured exposure-mediator confounding, ii) no unmeasured exposure-outcome confounding, iii) no unmeasured mediator-outcome confounding, iv) correct specification of the parametric models. We believe that we included the main exposure-mediator-outcome confounders (sex, year of birth, Health Board, ethnicity) and additional mediator-outcome confounders (maternal age, parity), which should reasonably capture biological, geographical and temporal sources of confounding, so are hopeful that assumptions i), ii) and iii) hold. All covariates entered the models as categorical variables so there was limited scope for model misspecification and we found no evidence of exposure-mediator or exposure-confounder interactions in any models, so these were not included (assumption iv)).

For continuous outcomes, in the absence of intermediate confounding (but with the above assumptions holding) the direct and indirect effects estimated under the traditional mediation analysis are consistent estimates of the NDE and NIE [61]. When considering height at age 4.5 years the estimated extent of mediation was greater under the traditional mediation analysis than under the counterfactual-based mediation analysis. This suggests that the observed differences in the results are due to intermediate confounding by maternal age and parity, which is correctly controlled for only in the counterfactual-based mediation analysis. This approach thus provides the more appropriate estimate of the extent of mediation.

Comparison of the results relating to overweight at age 4.5 years between the two analysis approaches is less straightforward. Even if the above assumptions hold and there is no intermediate confounding then the ‘direct effect’ and ‘indirect effect’ estimated under the traditional mediation analysis are not consistent estimates of the NDE and NIE due to the non-collapsibility of the OR [62]. It has recently been shown that the traditional mediation analysis will provide an overestimate (in absolute terms) of the NDE, leading to a corresponding bias in the estimated NIE [44]. In the present study we found the traditional mediation analysis to give an overestimate of the direct effect relative to the counterfactual-based mediation analysis, but it is not possible to distinguish between bias due to the non-collapsibility of the OR and bias due to incorrect control of intermediate confounding.

### Previous studies

Our finding of shorter childhood stature in more disadvantaged socioeconomic groups is in line with many previous studies [5–10], as is our observation of greater overweight in more disadvantaged socioeconomic groups [7, 8, 17, 18, 20–27, 29].

Although much previous research has suggested birth weight as a plausible mediator of the effect of social disadvantage on height or overweight, few studies have explicitly examined this mediation. Armstrong et al [35] used data from the CHSP Pre-school and analysed health records of 74,500 children aged 3–4 years in 1998–99. In comparing the most deprived (based on area-level Carstairs Deprivation Category) with least deprived, the odds ratio for obesity was 1.30 (95% CI: 1.05, 1.60), and when adjusting for birth weight the adjusted OR increased to 1.43 (95% CI: 1.16, 1.77), giving a proportion mediated of 0.21. Considering the differences in analysis approach, these results are very similar to those in the present study. However, the analysis of Armstrong et al [35] does not correctly account for intermediate confounding.

### Representativeness and generalisability

The CHSP Pre-School was implemented in different years in different Health Boards (mean 1994, range 1991 to 2000), meaning that not all births between 1991 and 2001 were eligible to have all childhood anthropometric measurements taken. Those measurements which could not be observed as they preceded the implementation of the CHSP Pre-School are systematically missing. The growth models required a minimum of one observation so that as many children as possible contributed to the analysis, with information borrowed from other children with more complete growth data. The lack of evidence for an interaction between Health Board and any of the exposures with respect to either of the outcomes suggests that our results remain representative of the target population.

Although the analyses considered relatively recent births (1991–2001), the fast-changing nature of the childhood obesity epidemic may mean that the findings are less applicable to current births. Further research is required in more recent cohorts.

### Strengths and limitations

There are many strengths to this analysis. The large sample size meant that we could estimate effects precisely. Although the analysis sample was a relatively small proportion of the overall sample the main reason for this (phased implementation of the CHSP Pre-School, described above) should not result in a biased sample–as borne out by the similarity of distributions of variables between the analysis sample and the overall sample. The data were from reliable sources (Census and birth records as part of the SLS, along with linked health data from maternity and CHSP Pre-School records).

There were also some limitations to the analysis. Although we included the main confounders and intermediate confounders, there may be others which we were unable to fully capture, potentially leading to bias. Potential unmeasured confounders could include maternal birth weight, parental anthropometrics, maternal lifestyle factors, and maternal morbidity. There may also be residual confounding due to measurement error or lack of granularity in those confounders that we did include.

Some of the variables included in the analyses may be subject to measurement error. This is a particular concern for synthetic income, for which error may be due to either the observed data on occupation or to the model from which this is converted into synthetic income. Such measurement error would in general lead to attenuation of the association between synthetic income and height or overweight, which may lead to some bias in the estimated direct and indirect effects.

Including a relatively small number of subjects with as few as a single growth measurement, allowing us to retain a larger sample size and reduce the potential for selection bias, may lead to mischaracterisation of the growth trajectories in those individuals. However, down-weighting these subjects in the traditional mediation analysis made very little difference (relative to the unweighted analysis) suggesting that the inclusion of subjects with fewer data points does not significantly affect the results (and certainly not the conclusions) of the study.

When using a two stage modelling approach (e.g. growth modelling followed by mediation analysis using the derived growth outcomes) one should appropriately propagate uncertainty in estimates from the first stage into the second stage model. In the traditional mediation analysis we weighted by the inverse of the approximate variance of the estimated growth outcomes to account for between-subject differences in their precision. While such weighting deals with relative variability in precision it does not allow for complete propagation of the first stage uncertainty, so the precision of the estimated effects may be somewhat overestimated in both analyses. Solutions include bootstrapping the whole process and joint modelling [63], but these may not be practicable for analyses using G-computation. Further research is required in this area.

## Conclusions

In this large-scale representative sample of the Scottish population we have elucidated the mediatory role of birth weight in the relationship between socioeconomic disadvantage and childhood growth in terms of height and overweight, and highlighted the importance of correctly accounting for intermediate confounding in mediation analyses. We found a strong, graded, negative effect of socioeconomic disadvantage on height which was mediated to some extent by birth weight and a strong, graded, positive direct effect of socioeconomic disadvantage on overweight which was partly masked by inconsistent mediation by birth weight.

Our findings suggest that higher birth weight in more disadvantaged groups may be associated with reduced social inequalities in height but also with increased inequalities in overweight.

## Supporting Information

S1 Appendix Growth modelling. (PDF)

S2 Appendix Total causal effect, natural direct effect and natural indirect effect on the odds ratio scale. (PDF)

S3 Appendix The G-computation procedure. (PDF)

S1 Table Growth data availability by age, Scottish Longitudinal Study, United Kingdom, 1991–2001. (PDF)

S2 Table Growth data availability by subject, Scottish Longitudinal Study, United Kingdom, 1991–2001. (PDF)

S3 Table Distributions of explanatory variables for study members included in the analysis and the dataset overall, Scottish Longitudinal Study, United Kingdom, 1991–2001. (PDF)

S4 Table Distributions of height and overweight at age 4.5 years by explanatory variable, Scottish Longitudinal Study, United Kingdom, 1991–2001. (PDF)
