# Supplementary material for: Pathways between Socioeconomic Disadvantage and Childhood Growth in the Scottish Longitudinal Study, 1991–2001
Source: PLoS One. 2016 Oct 13;11(10):e0164853. doi: 10.1371/journal.pone.0164853 (PMC5063393; doi:10.1371/journal.pone.0164853)
Supplement: S1 Appendix — (PDF) [file pone.0164853.s001.pdf]

Growth modelling

The repeated measurements of height and weight were modelled separately in males and females using mixed effects Berkey-Reed models (Berkey 1987; Goldstein 2011) which have been shown to provide a good fit to growth data in similar settings (Pizzi 2014):

$$y_{ij} = (\beta_0 + \beta_{0i}) + (\beta_1 + \beta_{1i})x_{ij} + \beta_2 log(x_{ij}) + \beta_3 \left(\frac{1}{x_{ij}}\right) + \varepsilon_{ij}$$

where y_{ij} is the height or weight of subject i at age x_{ij}, β_0 and β_{0i} are, respectively, the fixed and random terms for the intercept, β_1 and β_{1i} are, respectively, the fixed and random terms for the coefficient for x_{ij}, β_2 is the fixed coefficient for $log(x_{ij})$ and β_3 is the fixed coefficient for $\left(\frac{1}{x_{ij}}\right)$. β_{0i} and β_{1i} are assumed to be jointly normally distributed with means 0 and variance covariance matrix Σ, given x, and ε_{ij} is an error term, assumed to be independently and normally distributed given x, β_{0i} and β_{1i}, with variance allowed to vary across measurement occasions. Further random effects could not be included due to the relatively small number of repeated measurements per child. We used an unstructured variance-covariance matrix Σ for the random effects, meaning that the random intercept and slope terms could be correlated.

All subjects with at least one valid growth measurement were included in the modelling, assuming missingness was at random (Little 2002). The fitted models were used to predict subject-specific height (cm) and weight (kg) at age 4.5 years. The approximate variance of each predicted height and weight value was derived as a function of the estimated variances

of the random terms (variance of random intercept + 4.5^2 x variance of random age term). Predicted BMI at age 4.5 years was also derived from the relevant predicted height and weight values (weight (kg)/height $(m)^2$). The age- and sex-specific international overweight cut-offs (17.47 kg/m^2 for males, 17.19 kg/m^2 for females) of Cole et al (2000) were then used to define overweight/obese at age 4.5 years (a binary variable, hereafter denoted 'overweight')) from the predicted BMI. Predicted height (cm) and predicted overweight status were the outcomes of interest to be used in the second stage.

References

Berkey CS, Reed RB. A model for describing normal and abnormal growth in early childhood. Hum Biol. 1987; 59: 973-87.

Cole TJ, Bellizzi MC, Flegal KM, Dietz WH. Establishing a standard definition for child overweight and obesity worldwide: international survey. BMJ. 2000; 320: 1240-3.

Goldstein H. Multilevel Statistical Models. Chichester: John Wiley & Sons; 2011.

Little RJA, Rubin DB. Statistical Analysis With Missing Data. New York: Wiley; 2002.

Pizzi C, Cole TJ, Corvalan C, Dos Santos Silva I, Richiardi L, De Stavola BL. On modelling early life weight trajectories. J R Stat Soc Series A (Statistics in Society). 2014; 177(2): 371–96.
