# Supplementary material for: Pathways between Socioeconomic Disadvantage and Childhood Growth in the Scottish Longitudinal Study, 1991–2001
Source: PLoS One. 2016 Oct 13;11(10):e0164853. doi: 10.1371/journal.pone.0164853 (PMC5063393; doi:10.1371/journal.pone.0164853)
Supplement: S2 Appendix — (PDF) [file pone.0164853.s002.pdf]

Total causal effect, natural direct effect and natural indirect effect on the odds ratio scale

Let X_i be socioeconomic disadvantage, Y_i be (binary) overweight at age 4.5 years, M_i be birth weight, C_i be the set of baseline confounders (sex, year of birth, Health Board and ethnicity) and L_i be the set of intermediate confounders (maternal age and parity). Let $Y_i(x)$ be the value that Y_i would take if X_i had been set (possibly counter to fact) to the value x, $Y_i(x,m)$ be the value that Y_i would take if X_i and M_i had been set to the values x and m, and $M_i(x)$ be the value that M_i would take if X_i had been set to the value x.

The total causal effect (TCE) of X on Y, conditional on $C = c$, expressed as an odds ratio (OR) comparing $X = x^*$ to $X = x$ is

$$\mathrm{TCE}^{\mathrm{OR}}(c,x,x^*) = \frac{E(Y_i(x^*) \mid C_i = c) \;/\; (1 - E(Y_i(x^*) \mid C_i = c))}{E(Y_i(x) \mid C_i = c) \;/\; (1 - E(Y_i(x) \mid C_i = c))},$$

the natural direct effect (NDE) of X on Y, conditional on $C = c$, expressed as an OR comparing $X = x^*$ to $X = x$ is

$$\mathrm{NDE}^{\mathrm{OR}}(c,x,x^*) = \frac{E(Y_i(x^*, M_i(x)) \mid C_i = c) \;/\; (1 - E(Y_i(x^*, M_i(x)) \mid C_i = c))}{E(Y_i(x, M_i(x)) \mid C_i = c) \;/\; (1 - E(Y_i(x, M_i(x)) \mid C_i = c))},$$

and the natural indirect effect (NIE) of X on Y, conditional on $C = c$, expressed as an OR comparing $X = x^*$ to $X = x$ is

$$\text{NIEOR}(c, x, x^*) = \frac{E(Y_i(x^*, M_i(x^*)) \mid C_i = c) \;/\; (1 - E(Y_i(x^*, M_i(x^*)) \mid C_i = c))}{E(Y_i(x^*, M_i(x)) \mid C_i = c) \;/\; (1 - E(Y_i(x^*, M_i(x)) \mid C_i = c))}.$$
