# Supplementary material for: Pathways between Socioeconomic Disadvantage and Childhood Growth in the Scottish Longitudinal Study, 1991–2001
Source: PLoS One. 2016 Oct 13;11(10):e0164853. doi: 10.1371/journal.pone.0164853 (PMC5063393; doi:10.1371/journal.pone.0164853)
Supplement: S3 Appendix — (PDF) [file pone.0164853.s003.pdf]

The G-computation procedure

If it helpful to consider the total causal effect (TCE), natural direct effect (NDE) and natural indirect effect (NIE) in terms of the hypothetical worlds which they compare. For simplicity we initially consider only a binary exposure variable, but the following descriptions extend in a natural way to categorical exposure variables.

The TCE is a comparison of two hypothetical worlds: in the first everybody is exposed and in the second nobody is exposed. The NDE is also a comparison of two hypothetical worlds: in the first everybody is exposed and in the second nobody is exposed; in both worlds the mediator is set to the value it would naturally take in the absence of exposure. The NDE thus captures the effect of the exposure on the outcome via pathways that do not involve the mediator. The NIE is again a comparison of two hypothetical worlds: in the first the mediator is set to the value it would take in the presence of exposure and in the second the mediator is set the value it would take in the absence of exposure; in both worlds the exposure is set to be present. The NIE therefore captures the effect of the exposure on the outcome through the mediator. These definitions extend in a natural way to categorical exposure variables, as in the present study, with each separate estimate for a non-baseline level of the variable relative to the baseline level of the variable defined in the above manner.

Estimation for the counterfactual-based mediation analysis was performed via parametric G-computation using Monte Carlo simulation (Robins 1986; Daniel 2011; Daniel 2013).

The first stage of the G-computation procedure is to model the relationships between the variables in the observed data. Taking the analysis regarding height age 4.5 years and mother's education as an example:

- Maternal age was modelled using multinomial logistic regression as a function of age 4.5 years mother's education and the background confounders (sex, year of birth, Health Board and ethnicity);
- Maternal parity was modelled using multinomial logistic regression as a function of mother's education, the background confounders and maternal age;
- Birth weight was modelled using multinomial logistic regression as a function of mother's education, the background confounders, maternal age and maternal parity; and
- Height age 4.5 years was modelled using linear regression as a function of mother's education, the background confounders, maternal age and maternal parity, and birth weight.

Using the resultant models we then simulated the outcomes for each of the hypothetical worlds compared in the definitions of the TCE, NDE and NIE. Simulations were carried out forward in time, starting by modelling maternal age given mother's education and the background confounders, and proceeded through the models in the order above. By setting mother's education to each level in turn we simulated height in the hypothetical worlds compared in the TCE. By setting mother's education to its baseline value we simulated the value birth weight would naturally take in the absence of exposure. This was then used, with mother's education set to each level in turn, to simulate height in the hypothetical worlds

compared in the NDE. Finally, we simulated the value of birth weight at each level of mother's education. These were used, with mother's education set to each non-baseline level in turn, to simulate the hypothetical worlds compared in the NIE. Causal inference was then pursued by comparing the average simulated outcomes in the hypothetical worlds.

References

Daniel RM, De Stavola BL, Cousens SN. gformula: Estimating causal effects in the presence of time-varying confounding or mediation using the g-computation formula. The Stata Journal. 2011; 11: 479-517.

Daniel RM, Cousens SN, De Stavola BL, Kenward MG, Sterne JA. Methods for dealing with time-dependent confounding. Stat Med. 2013; 32: 1584-618.

Robins J. A new approach to causal inference in mortality studies with a sustained exposure period - application to control of the healthy worker survivor effect. Mathematical Modelling. 1986; 7: 1393–512.
