# Supplementary material for: Pathways between Socioeconomic Disadvantage and Childhood Growth in the Scottish Longitudinal Study, 1991–2001
Source: PLoS One. 2016 Oct 13;11(10):e0164853. doi: 10.1371/journal.pone.0164853 (PMC5063393; doi:10.1371/journal.pone.0164853)
Supplement: S1 Table — (PDF) [file pone.0164853.s004.pdf]

Table 1. Growth data availability by age, Scottish Longitudinal Study, United Kingdom, 1991-2001.

Approximate age	Number of available measurements (% of total number of subjects included in growth model[a])	
	Height	Weight
6-8 weeks	14,887 (60.3)	14,604 (59.3)
8-9 months	15,638 (63.3)	15,615 (63.4)
21-24 months	13,225 (53.5)	13,347 (54.2)
39-42 months	19,004 (76.9)	19,056 (77.4)
48 months	12,978 (52.5)	12,869 (52.2)

[a]24,703 for height and 24,632 for weight.

Source: Scottish Longitudinal Study.
