# Supplementary material for: Pathways between Socioeconomic Disadvantage and Childhood Growth in the Scottish Longitudinal Study, 1991–2001
Source: PLoS One. 2016 Oct 13;11(10):e0164853. doi: 10.1371/journal.pone.0164853 (PMC5063393; doi:10.1371/journal.pone.0164853)
Supplement: S2 Table — (PDF) [file pone.0164853.s005.pdf]

Table 2. Growth data availability by subject, Scottish Longitudinal Study, United Kingdom, 1991-2001.

Number of valid measurements	Number (%) of subjects	
	Height	Weight
1	4378 (17.7)	4382 (17.8)
2	4730 (19.1)	4780 (19.4)
3	5380 (21.8)	5266 (21.4)
4	5825 (23.6)	5738 (23.3)
5	4390 (17.8)	4466 (18.1)
Total	24,703	24,632

Source: Scottish Longitudinal Study.
