# Supplementary material for: Pathways between Socioeconomic Disadvantage and Childhood Growth in the Scottish Longitudinal Study, 1991–2001
Source: PLoS One. 2016 Oct 13;11(10):e0164853. doi: 10.1371/journal.pone.0164853 (PMC5063393; doi:10.1371/journal.pone.0164853)
Supplement: S3 Table — (PDF) [file pone.0164853.s006.pdf]

Table 3. Distributions of explanatory variables for study members included in the analysis and the dataset overall, Scottish Longitudinal Study, United Kingdom, 1991-2001.

Variable	Analysis sample[a] (n = 16,628) N	n (%)	Sample overall (n = 43,286) N	n (%)
Mother's education	15,031		27,198	
No qualifications		2,330 (15.5)		4,698 (17.3)
GCSE or equivalent		5,355 (35.6)		9,696 (35.6)
A-level of equivalent		3,879 (25.8)		6,712 (24.7)
Degree or equivalent		3,467 (23.1)		6,092 (22.4)
Scottish Index of Multiple Deprivation quarter	16,588		34,712	
1 (Most deprived)		3,970 (23.9)		8,747 (25.2)
2		4,291 (25.9)		8,721 (25.1)
3		3,967 (23.9)		8,639 (24.9)
4 (Least deprived)		4,360 (26.3)		8,605 (24.8)
Synthetic income quarter	16,627		34,912	
1 (lowest)		4,552 (27.4)		10,476 (30.0)
2		4,618 (27.8)		9,858 (28.2)
3		4,429 (26.6)		8,666 (24.8)
4 (highest)		3,028 (18.2)		5,912 (16.9)
Sex	16,628		43,262	
Male		8,578 (51.6)		22,172 (51.3)
Female		8,050 (48.4)		21,090 (48.7)
Year of birth	16,628		43,286	
1991-1994		5,323 (32.0)		17,377 (40.1)
1995-1998		5,642 (33.9)		11,871 (27.4)
1999-2001		5,663 (34.1)		14,038 (32.4)
Health Board	16,628		26,537	
Ayrshire & Arran		1,126 (6.8)		1,750 (6.6)
Borders		483 (2.9)		698 (2.6)
Argyll & Clyde		1,959 (11.8)		3,046 (11.5)
Fife		1,441 (8.7)		2,164 (8.2)
Greater Glasgow		3,141 (18.9)		5,618 (21.2)
Lanarkshire		2,499 (15.0)		3,877 (14.6)
Lothian		3,295 (19.8)		5,309 (20.0)
Tayside		1,568 (9.4)		2,279 (8.6)
Forth Valley		905 (5.4)		1,316 (5.0)
Dumfries & Galloway		211 (1.3)		480 (1.8)
Ethnicity	16,628		28,314	

White		16,174 (97.3)		27,446 (96.9)
Non-white		454 (2.7)		868 (3.1)
Birth weight (kg)	16,628		32,452	
<2.50		807 (4.9)		1,761 (5.4)
2.50-2.99		2,450 (14.7)		4,972 (15.3)
3.00-3.49		5,959 (35.8)		11,732 (36.2)
3.50+		7,412 (44.6)		13,987 (43.1)
Mother's age (years)	16,628		34,889	
<20		1,123 (6.8)		2,744 (7.9)
20-24		2,869 (17.3)		6,897 (19.8)
25-29		5,427 (32.6)		11,278 (32.3)
30-34		5,031 (30.3)		9,720 (27.9)
35+		2,178 (13.1)		4,250 (12.2)
Parity	16,628		32,432	
0		7,678 (46.2)		14,656 (45.2)
1		5,879 (35.4)		11,311 (34.9)
2+		3,071 (18.5)		6,465 (19.9)

[a]Complete data on birth weight, all the potential confounders and one or more of the socioeconomic disadvantage measures.

Source: Scottish Longitudinal Study.
