# Supplementary material for: Pathways between Socioeconomic Disadvantage and Childhood Growth in the Scottish Longitudinal Study, 1991–2001
Source: PLoS One. 2016 Oct 13;11(10):e0164853. doi: 10.1371/journal.pone.0164853 (PMC5063393; doi:10.1371/journal.pone.0164853)
Supplement: S4 Table — (PDF) [file pone.0164853.s007.pdf]

Table 4. Distributions of height and overweight at age 4.5 years by explanatory variable, Scottish Longitudinal Study, United Kingdom, 1991-2001.

	Height			Overweight		
	Mean (SD) height (cm)		*P* overall	n (%) overweight		*P* overall
Variable	Males	Females	(*P* trend)[A]	Males	Females	(*P* trend)[A]
Mother's education						
No qualifications	106.3 (4.2)	105.5 (4.2)	<0.001	140 (12.0)	183 (15.9)	0.11
GCSE or equivalent	106.8 (4.1)	106.0 (4.0)		288 (10.5)	403 (15.7)	(0.04)
A-level of equivalent	107.4 (3.9)	106.3 (4.0)		242 (12.1)	284 (15.3)	
Degree or equivalent	107.4 (4.0)	106.4 (4.1)		175 (9.7)	237 (14.4)	
Scottish Index of Multiple Deprivation quarter						
1 (most deprived)	106.5 (4.1)	105.5 (4.1)	<0.001	244 (12.1)	298 (15.6)	0.05
2	106.9 (4.2)	106.0 (4.0)	(<0.001)	242 (10.9)	325 (15.9)	(0.02)
3	107.2 (4.0)	106.1 (4.1)		224 (11.0)	299 (15.7)	
4 (least deprived)	107.3 (3.9)	106.4 (4.1)		231 (10.3)	293 (13.9)	
Synthetic income quarter						
1 (lowest)	106.6 (4.1)	105.7 (4.0)	<0.001	268 (11.5)	327 (15.1)	0.78
2	106.7 (4.1)	105.9 (4.1)		262 (11.0)	337 (15.2)	(0.64)
3	107.3 (4.1)	106.3 (4.0)		241 (10.7)	325 (15.2)	
4 (highest)	107.3 (4.0)	106.3 (4.2)		171 (11.0)	229 (15.7)	
Sex						
Male	107.0 (4.1)	-	<0.001	942 (11.0)	-	<0.001
Female	-	106.0 (4.1)		-	1218 (15.2)	

Year of birth						
1991-1994	106.7 (4.1)	105.9 (4.1)	<0.001	301 (11.1)	373 (14.7)	0.12
1995-1998	107.1 (4.0)	106.2 (4.0)		292 (10.2)	425 (15.5)	(0.08)
1999-2001	107.1 (4.1)	106.0 (4.1)		349 (11.9)	420 (15.4)	
Health Board						
Ayrshire & Arran	107.1 (4.1)	106.4 (4.0)	0.001	60 (10.7)	77 (14.3)	0.53
Borders	107.4 (4.3)	106.5 (4.3)		24 (10.3)	39 (15.6)	
Argyll & Clyde	107.0 (4.2)	106.1 (4.0)		117 (11.3)	148 (16.3)	
Fife	106.8 (3.8)	105.6 (4.1)		92 (11.7)	108 (16.7)	
Greater Glasgow	107.1 (4.0)	106.1 (4.1)		182 (11.5)	217 (14.2)	
Lanarkshire	106.8 (4.1)	106.0 (3.9)		139 (11.0)	184 (15.0)	
Lothian	106.8 (4.0)	106.0 (4.1)		172 (10.2)	265 (16.6)	
Tayside	106.9 (4.0)	105.8 (4.1)		89 (10.5)	92 (12.8)	
Forth Valley	107.2 (4.5)	106.3 (4.1)		57 (13.1)	74 (16.4)	
Dumfries & Galloway	107.5 (3.6)	105.8 (4.5)		10 (9.9)	14 (13.0)	
Ethnicity						
White	107.0 (4.1)	106.0 (4.1)	0.25	922 (11.1)	1196 (15.3)	0.001
Non-white	107.1 (4.2)	106.4 (4.7)		20 (7.8)	22 (11.3)	
Birth weight (kg)						
<2.50	104.5 (4.3)	103.3 (4.5)	<0.001	28 (7.3)	33 (7.9)	<0.001
2.50-2.99	105.1 (4.0)	104.5 (3.9)		86 (7.8)	141 (10.6)	(<0.001)
3.00-3.49	106.5 (3.8)	105.8 (3.8)		232 (8.3)	438 (14.0)	

3.50+	107.9 (3.9)	107.2 (3.9)		596 (14.0)	606 (19.5)	
Mother's age (years)						
<20	106.6 (3.9)	105.7 (4.1)	<0.001	72 (12.1)	73 (14.0)	0.82
20-24	106.5 (4.2)	105.6 (4.1)		156 (10.8)	210 (15.0)	(0.90)
25-29	107.1 (4.1)	106.1 (4.0)		305 (10.9)	410 (15.9)	
30-34	107.1 (4.0)	106.2 (4.0)		274 (10.5)	347 (14.4)	
35+	107.2 (4.0)	106.2 (4.4)		135 (12.5)	178 (16.4)	
Parity						
0	107.2 (4.1)	106.3 (4.1)	<0.001	425 (10.8)	574 (15.5)	0.75
1	106.9 (4.0)	105.8 (4.0)	(<0.001)	345 (11.3)	423 (15.1)	(0.64)
2+	106.4 (4.1)	105.5 (4.1)		172 (11.0)	221 (15.0)	

[A]*P*-values from Wald tests following the fitting of linear or logistic regression models of height or overweight, respectively, on each explanatory variable, adjusted for sex, weighted by the inverse of the approximate variance of the outcome, and using robust standard error estimation. *P*-value for trend presented for each ordered categorical explanatory variable, unless there was evidence of non-linearity. No evidence of interaction by sex.

Source: Scottish Longitudinal Study.
